# The Role of Stress Proteins in the Study of Allostatic Overload in Birds: Use and Applicability to Current Studies in Avian Ecology

**DOI:** 10.1100/tsw.2007.242

**Published:** 2007-09-28

**Authors:** Garth Herring, Dale E. Gawlik

**Affiliations:** Department of Biological Sciences, Florida Atlantic University, 777 Glades Road, Boca Raton, FL 33431, USA

**Keywords:** corticosterone, glucocorticoids, heat shock protein, HSP, physiology, stress protein, vertebrate stress response, birds

## Abstract

Stress proteins offer a measure of stress in birds at the cellular level that are an alternative to the glucocorticoids. Stress proteins are not biased by handling stress, the increase in stress proteins lasts longer than with other measures (e.g., corticosterone), and, therefore, they may be a more appropriate measure of long-term or chronic stress. However, caution should be practiced when using stress proteins because the level of stress needed to elicit a response may be higher than with corticosterone. Stress proteins have only recently been used to measure the response to competition, food limitation, growth, and parasitism in birds. In other taxa, the stress proteins have been used to measure genetic stress, temperature, toxins, UV radiation, and physical activity. Stress proteins increase the options available to avian ecologists for understanding how avian species respond to changes in the environment.

In avian ecology studies, the physiological condition of an individual is often induced by changes in their environment and can be measured by endocrinological parameters. These nonlethal techniques allow ecologists to assess the physiological condition of species simply through the collection of blood or fecal material. Additional advantages of these techniques are that the same animal can be measured repeatedly throughout time and space (e.g., breeding season), and they can be applied to endangered species. One of the most popular approaches to studying the physiological condition of avian species to an environmental stimulus or stressor is the measurement of corticosterone (CORT), in plasma or fecal samples. There are, however, a number of issues with using CORT as a metric of stress in birds and less-recognized measures of stress, such as the stress proteins, could provide an alternative, possibly improving our understanding of avian responses to stress.

In this paper, we briefly review the conventional use of CORT in avian physiology/ecology studies, detailing the breadth of its use and limitations, including potential problems with using CORT as a measure of stress in birds. We then introduce the stress proteins, provide background on the role they play during stress responses, detail their use across research disciplines and recent use in avian ecology, and lastly examine the limitations of using stress proteins in avian ecology. With this paper, we hope to generate further interest in the use of stress proteins in avian ecology; in particular, experimental approaches that allow a better understanding of the role stress proteins play in birds seasonally, their behavior during normal conditions (nonstress periods), and their response to stress in the absence of additional factors that often reduce our ability to understand ecological responses.

## DEFINING STRESS

Minor fluctuation of CORT in birds as well as other vertebrates is relatively common due to the highly variable environments in which they live. Most species are adapted to these variable environments and can adjust their physiology in order to cope[1,2,3]. Critical to the overall question being asked of how a species responds to a stressor is the definition of stress. Romero[3] defined stress as a noxious stimulus that results in a suite of physiological and behavioral coping mechanisms, with long-term chronic stress resulting in overstimulation of the coping mechanisms. Often in the presence of unpredicted, highly variable fluctuations of negative stimuli, chronic stress will become a factor that could result in negative impacts on an individual or population[1,3]. Ewen and Wingfield[1] proposed that we begin to frame our discussions of stress within the concept of allostasis, the maintenance of physiological homeostasis during times of stress. Allostatic load is how hard an individual must work in order to maintain that balance, and allostatic overload is when the energy requirements to maintain the balance exceed the capacity of the animal.

## TRADITIONAL APPROACH TO MEASURING STRESS: CORTICOSTERONE

CORT serves as a physiological signal to a bird to modify its behavior and/or metabolism to deal with potentially adverse environmental conditions. When the cortex of the brain detects a stressor, a neural signal is sent to the hypothalamus. The hypothalamus then sends a hormone signal to the pituitary gland, resulting in the pituitary gland sending a signal to the adrenal or inter-renal gland to release CORT. After the stressor has been alleviated, a negative feedback loop shuts off the hypothalamus-pituitary-adrenal (HPA) pathway leading to the CORT release. If the stressor persists and CORT remains elevated, the negative feedback loop will cease to function and destructive chronic effects may begin (e.g., reproductive failure) as allostatic overload occurs.

At lower levels, CORT can stimulate foraging activity and lipogenesis[4,5]. Under conditions of food scarcity, CORT stimulates gluconeogenesis, shifting catabolism away from fats towards proteins[4,5]. Chronic elevated levels of CORT can reduce the fitness of individuals via immunosuppression and reproductive failure, resulting in harmful effects at various levels[6,7,8]. Fecal CORT levels reflect residue levels of plasma CORT; however, rapid and extensive metabolization before excretion results in lower overall levels than circulating levels[9].

While CORT has played an important role in understanding how wild and captive birds respond to environmental stimuli (e.g., nutritional conditions[10]), it has a major limitation in that CORT production can be stimulated by capture and handling stress. Therefore, unless plasma samples are collected within the first 2–3 min, they will often be biased high, reflecting the stress induced by the researcher and not the basal level of CORT, which is of interest[11]. Measuring fecal CORT is one approach to reduce bias from capture and handling stress because elevation of fecal CORT does not occur for 2–12 h after capture[12,13,14,15]. However, fecal CORT measurement also has limitations. This steroid is prone to degradation when fecal samples are frozen for storage and as they degrade, the concentration of cross-reacting metabolites can change, potentially giving the impression that hormone levels increased since sampling[14,15]. Often, fecal samples will include both feces and uric acid; however, CORT may be excreted at different levels in feces and uric acid resulting in an increase in sample variance[14,15,16,17].

Additionally, there are two types of CORT that can be measured and the scientific community has not agreed on a standard. Free CORT is available to interact with receptors and can result in a response to the stressor, whereas total CORT is bound to a carrier protein and is not available to receptors[18,19]. Breuner and Orchinik[18] suggested that free CORT might be the more important parameter to measure because it interacts with receptors; however, most studies to date have measured total CORT. It has been shown that different species may have opposite relationships between free and total CORT[19,20,21], and, therefore, measuring total CORT in some species may be sufficient, whereas in others it may not. Previous studies found that free CORT in house sparrows (*Passer domesticus*) increased when the individual was stressed, while total CORT remained relatively constant[18,19]. Other studies[20] have found a different trend, with free CORT and total CORT having similar circulating levels. Understanding the response of free CORT and total CORT should be a consideration of future studies using CORT as a response to a stressor. It is also important for researchers to acknowledge which CORT type they have measured, so that comparisons are made properly[18,19].

## ALTERNATIVE APPROACH TO MEASURING STRESS: STRESS PROTEINS

Stress proteins are a group of highly conserved, intracellular polypeptides found in all organisms from bacteria to humans, indicating a crucial role in cellular survival[22]. They function as molecular chaperones for proteins within cells and present a major molecular barrier to alterations in cellular homeostasis[22,23]. The primary role of stress proteins is to limit the interactions of unfolded proteins during the early stages of their synthesis by identifying and binding to exposed proteins. The unfolded protein is stabilized, protecting it from damage from destructive situations, and can then be restored or is tagged for destruction[23,24]. Most stress proteins are constitutively expressed, supporting normal protein folding and scouring the cell for damaged proteins. However, some stress proteins are transcriptionally up-regulated in a cell on exposure to a variety of stressors[22]. Heat shock factor (HSF) is a transcription factor that acts as a regulator for heat shock proteins (HSP)[22].

Under normal cellular conditions, stress proteins are involved in routine cellular protection; however, their molecular chaperone role is amplified during periods of increased stress in order to regulate cell protein damage[22]. There are numerous families of stress proteins, characterized by their molecular weights in kilodaltons, which have been reviewed in detail by Parsell and Lindquist[25] and Feder and Hoffman[26]. The most commonly studied stress proteins in wild animals are HSP60 and HSP70[24]. Additional families of stress proteins may prove to be beneficial when measuring the response of birds to stress, but little work has been done as yet to assess the validity of using those other families.

Stress proteins are up-regulated in response to a variety of stressors in many different species and taxas, including temperature[26,27,28], heavy metals[29,30], parasites[31,32], nutritional stress[33], hypoxia[34], anoxia[35], high density[36], pesticides[37,38], food limitation, UV radiation, physical activity, and genetic stresses[24,26,39]. In comparison to CORT, stress proteins may be a more appropriate metric for detecting long-term or chronic exposure to stress resulting in allostatic overload[24,40] because it takes several hours to show up in blood cells and remains there longer than CORT[41]. While stress proteins can serve as metrics of stress from a range of variables, they have only recently begun to be used in avian ecology.

Several recent avian ecology studies have successfully used stress proteins to link environmental stress to physiological factors measured in birds. Merino et al.[32] examined how stress proteins responded to parasitism in nestling house martins (*Delichon urbica*), observing a negative relationship between chick growth and stress protein levels. These results suggest that house martins may have a limited amount of energy to expand on different nestling functions; the response of elevated stress proteins to increased parasites may reduce energy required for chick growth[32]. Moreno et al.[33] also found a negative relationship between stress proteins and chick growth parameters in pied flycatchers

(*Ficedula hypoleuca*). These authors observed that up to 80% of the variation in some growth parameters of pied flycatchers could be explained by stress proteins[33].

Martínez-Padilla et al.[41] found that stress proteins were an efficient estimator of competitive, nutritional, and parasite-mediated stress in nestling Eurasian kestrels (*Falco tinnunculus*). Smaller Eurasian kestrel nestlings had higher levels of stress proteins, indicative of sibling competition and food shortage[41]. Garamszegi et al.[41] examined the response of sexual traits to increased stress proteins in the collared flycatcher (*Ficedula albicollis*), observing that stress proteins may constrain the expression of some specific sexual traits. Male collared flycatchers that produced longer and more versatile songs had higher levels of stress proteins[42]. Bourgeon et al.[43] found that incubating female common eiders (*Somateria mollissima*) decreased their immunoglobulin corresponding to an increase in levels of HSP60 and HSP70. Additionally, CORT levels in eiders decreased during the incubation period, while stress proteins increased[43]. These authors contend that these results supported the theory that CORT does not regulate immunosuppression and, additionally, that stress proteins levels were not triggered by elevated levels of CORT[44]. Lastly, Tomás et al.[23] found that blood stress protein levels did not change within 8 h of sampling in the field when not frozen or centrifuged, a critical issue if collecting samples from wild birds, as in most avian ecology studies.

Common methods to measure the stress proteins include western blotting, slot blotting, radioimmunoassay (RIA), and enzyme-linked immunosorbent assay (ELISA)[44]. Lewis et al.[44] detailed the use of all methods available for the detection of stress proteins, noting that western blotting has been the most common method used to measure stress proteins to date. Dot slot blotting was suggested to be a simplified approach to measuring stress proteins, but ELISAs and RIAs provide more quantitative results[44]. Stress proteins are measurable in cell lysates, tissue extracts, red blood cells, and serum samples. Levels of stress proteins may differ between these sample types[44], so any comparisons made between studies or sample types should be limited to the response and not specific levels of stress proteins. Blood samples provide a reliable nonlethal approach to measuring stress proteins[33,39].

## CONCLUSIONS

When considering the use of stress proteins as a metric of environmental stress, careful consideration should be paid to which family of stress proteins is being measured. Individual families of stress proteins may respond differently to environmental stimuli stressors than other families; where one stress protein family increases in the presence of stress, another may remain constant. Martínez-Padilla et al.[41] observed a significant increase in stress protein 60 during the course of a breeding season in Eurasian kestrels, while stress protein 70 remained relatively constant. Moreno et al.[33] also found stress protein 60 levels to be negatively correlated with tarsus length and high heterophil/lymphocyte ratios, with no relationship to stress protein 70 levels. Stress proteins can also play an important role in normal cellular activities, so understanding which families are involved with specific cellular responses and what basal levels are is significant when interpreting the response to a stimuli, just as when using CORT.

One aspect of stress protein research that can greatly enhance our understanding of homeostatic and allostatic overload levels of stress proteins in different species of birds is if there are differences associated with season, reproductive status, sex, etc., and how those differences might be expressed with the different families of stress proteins and in different sample types. Those avian ecology studies that have used the stress proteins as a measure of response to environmental stress have largely been correlative to date. Conducting controlled experiments within aviaries could be a valuable approach to understanding the true response of the stress proteins during periods of nonstress, homeostasis, and allostatic overload, in the absence of other variables that can skew the interpretation of results in field studies (e.g., food abundance, temperature).

Recent research on fish (Silver Sea Bream *Sparus sarba*) demonstrated that cortisol (the CORT equivalent found in fishes) could act both as an anti- or a proapoptotic by either inducing or suppressing stress proteins[45]. These results suggest that understanding the relationship of CORT and stress proteins in birds should be studied to determine how they might differ in their response to stress and/or interact. Understanding the mechanisms behind the expression or transcriptional up-regulation of stress protein also remains poorly understood for avian species. These mechanisms are critical to understanding how apparently stressed individuals (e.g., elevated CORT) might still have lower levels of stress proteins and vice versa.

The use of stress proteins in avian ecology is still in its infancy, but shows promise for being a valuable alternative metric of environmental stress in avian ecology. Stress proteins provide another method to examine the physiological response of species to stressors, and could become routine metrics of physiological condition if avian ecologists become more aware of these options. As with using CORT in avian ecology studies of stress responses to environmental stimuli, the use of stress proteins requires a thorough understanding of a species' physiology, the stressor, and the stress protein measured. Many of these assumptions and issues have already been tested on other vertebrate groups, so our ability to utilize these proteins in avian ecology may not be as limited as perceived. Stress proteins may not prove to be the best measure of stress in all situations or in all species. They do, however, increase the options available to avian ecologists for understanding how species respond to changes in the environment and may enhance our ability to make more informed decisions regarding the conservation of birds.

## ACKNOWLEDGMENTS

Funding for research that led to conversations on the use of CORT and stress proteins that culminated in the writing of this manuscript was provided by the U.S. Fish and Wildlife Service. We thank Tylan Dean for facilitating the funding of this study. Heidi Herring, Sarah Milton, and one anonymous reviewer provided valuable comments on previous drafts of this manuscript.
